# Rituximab, plasma exchange and immunoglobulins: an ineffective treatment for chronic active antibody-mediated rejection

**DOI:** 10.1186/s12882-018-1057-4

**Published:** 2018-10-11

**Authors:** Gastón J Piñeiro, Erika De Sousa-Amorim, Manel Solé, José Ríos, Miguel Lozano, Frederic Cofán, Pedro Ventura-Aguiar, David Cucchiari, Ignacio Revuelta, Joan Cid, Eduard Palou, Josep M Campistol, Federico Oppenheimer, Jordi Rovira, Fritz Diekmann

**Affiliations:** 1 Department of Nephrology and Renal Transplantation, ICNU, Hospital Clinic, Barcelona, Spain; 2 Laboratori Experimental de Nefrologia i Trasplantament (LENIT), IDIBAPS, Barcelona, Spain; 3 Department of Pathology, Hospital Clinic, Barcelona, Spain; 4 Medical Statistics Core Facility, IDIBAPS, Hospital Clinic, Barcelona, Spain; 5 Biostatistics Unit, Faculty of Medicine, Autonomous University of Barcelona, Barcelona, Spain; 6 Apheresis Unit, Department of Hemotherapy and Hemostasis, IDIBAPS, Hospital Clinic, Barcelona, Spain; 7 Red de Investigación Renal (REDinREN), Madrid, Spain; 8 Department of Immunology, Hospital Clinic, Barcelona, Spain

**Keywords:** Kidney transplantation, Transplant glomerulopathy, Chronic active antibody-mediated rejection, Rituximab, Infections

## Abstract

**Background:**

Chronic active antibody-mediated rejection (c-aABMR) is an important cause of allograft failure and graft loss in long-term kidney transplants.

**Methods:**

To determine the efficacy and safety of combined therapy with rituximab, plasma exchange (PE) and intravenous immunoglobulins (IVIG), a cohort of patients with transplant glomerulopathy (TG) that met criteria of active cABMR, according to BANFF’17 classification, was identified.

**Results:**

We identified 62 patients with active c-aABMR and TG (cg ≥ 1). Twenty-three patients were treated with the combination therapy and, 39 patients did not receive treatment and were considered the control group. There were no significant differences in the graft survival between the two groups. The number of graft losses at 12 and 24 months and the decline of eGFR were not different and independent of the treatment. A decrease of eGFR≥13 ml/min between 6 months before and c-aABMR diagnosis, was an independent risk factor for graft loss at 24 months (OR = 5; *P* = 0.01). Infections that required hospitalization during the first year after c-aABMR diagnosis were significantly more frequent in treated patients (OR = 4.22; *P* = 0.013), with a ratio infection/patient-year of 0.65 and 0.20 respectively.

**Conclusions:**

Treatment with rituximab, PE, and IVIG in kidney transplants with c-aABMR did not improve graft survival and was associated with a significant increase in severe infectious complications.

**Trial registration:**

Agencia Española de Medicametos y Productos Sanitarios (AEMPS): 14566/RG 24161. Study code: UTR-INM-2017-01.

**Electronic supplementary material:**

The online version of this article (10.1186/s12882-018-1057-4) contains supplementary material, which is available to authorized users.

## Background

Chronic active antibody-mediated rejection (c-aABMR) is a major cause of renal allograft failure in kidney transplants [1, 2]. Transplant glomerulopathy (TG), one of the histological features of c-aABMR, results from continuous endothelial injury and repair processes, leading to pathological multi-layering of the glomerular basement membrane [3]. The prevalence of TG increases over time after transplantation and has been associated with reduced allograft outcomes, with a mean allograft survival of 2 years after diagnosis [2, 4–6].

Despite its clinical significance the available evidence on treatment of c-aABMR with TG is scarce. Similar to the treatment of active antibody-mediated rejection, many centers use combinations of plasma exchange (PE), immunoglobulin (IVIG) and rituximab (RTX) therapy for c-aABMR. Small and retrospective series of cases reported a slight improvement in patients with c-aABMR under IVIG and rituximab treatment [7–10]. In contrast, in two recent patients series, improvements of graft survival were not observed when comparing untreated with treated patients, whereas treated patients suffered a higher incidence of complications and adverse effects [11, 12]. However, untreated groups were small in both studies.

Recently in a randomized trial, the efficacy of rituximab and IVIG vs. placebo was tested in 24 patients with TG and DSA. There was no difference in eGFR decline. Unfortunately the study was underpowered, because the recruitment was stopped early due to low inclusion rate [13].

In the present study, we retrospectively reviewed 62 patients with c-aABMR and TG to determine the efficacy and safety of the combined therapy of RTX, PE, and IVIG.

## Methods

### Study population

We retrospectively reviewed our pathology database between 2006 and 2015, identified all patients with TG (cg ≥ 1 in the Banff histopathological classification) and re-evaluated them according to Banff 2017 classification criteria [3].

The inclusion criteria were the coexistence of c-aABMR TG with microvascular injury (MVI) ≥2 (g + ptc ≥ 2), with positive or negative C4d staining in peritubular capillaries and positive donor-specific antibodies (DSA). The patients with compatible histology, but negative DSA were included in the analysis as suspicious of c-aABMR. Also, TG plus positive C4d or TG with MVI =1 plus positive DSA were included. The presence of concomitant cellular rejection required at least g of 1.

The decisions to perform a renal biopsy and patient treatment were based on the clinical judgment at c-aABMR diagnosis.

Every patient who received treatment with RTX, IVIG, and PE after diagnosis of c-aABMR was included in the treatment group. Patients who did not receive RTX, IVIG neither PE, alone or in combination, were included in the control group.

PE was performed in Cobe Spectra or Spectra Optia separators (Terumo BCT, Lakewood, CO, USA) using 5% albumin (Albutein® 5%, Grífols, Spain) as a replacement solution. One plasma volume was exchanged in each session as previously reported [14].

The primary endpoint was graft survival. Secondary endpoints were the evolution of glomerular filtration rate and complications related to treatment.

The Institutional Ethics Committee approved the study. The registration number was 14,566/RG 24161 (Agencia Española del Medicamento y Producto Sanitario, AEMPS).

### Clinical data

The clinical characteristics, immunosuppression, and treatment were analyzed at the time of c-aABMR diagnosis and in the follow-up period. We assessed Charlson comorbidity index (CCI) at diagnosis of c-aABMR [15]. In accordance with other groups, CCI was unadjusted for age, and the minimum score of our patients was 0 (2 points for renal insufficiency were not added) [16–19].

We assessed renal function and proteinuria at c-aABMR diagnosis, 6 months before and 6, 12, 24 months after diagnosis and at the end of follow-up. Renal function was determined by serum creatinine and by estimated glomerular filtration rate (eGFR) using the Modification of Diet in Renal Disease (MDRD) formula [20]. Proteinuria was evaluated using the proteinuria/creatinine index [21].

Serum samples obtained at the moment of transplantation and rejection were screened for HLA class I and II antibodies using the Lifecodes LifeScreen Deluxe flow bead assay (Immucor, Stamford, CT, USA). Antibody specificities were determined using the Lifecodes Single Antigen bead assay (Immucor, Stamford, CT, USA) in patients with positive HLA antibodies.

Infections that required hospitalization at least 48 h during the first year after c-aABMR diagnosis were analyzed. All immediate adverse events (AE), after IVIG, PE and RTX infusion were registered.

### Statistical analysis

Data were described as mean with standard deviation (SD) or standard error of the mean (SEM), in case of graphical presentation for quantitative variables and as absolute and relative (%) frequencies for qualitative variables. Group comparisons between patients with or without the intake of rituximab were made by Fisher’s exact, t-test or Mann Whitney U test for independent groups. Kaplan-Meier and comparison between both groups were made using Log-Rank test, considering non-related death as censure. We performed a crude estimation of the effect of treatment on the evolution of eGFR by generalized estimating equation (GEE) model using an AR(1) matrix to estimate the intra-subject correlation. These models included treatment effect, time of follow-up and their interaction with treatment. GEE models of treatment effect were adjusted for confounding factors including infectious disease. As a useful clinical evaluation of prediction values of change of eGFR (Δ of change) from 6 months before to rejection, we proposed a cutoff using the Likelihood Ratio (LR^+^) defined as ratio sensitivity/(1-specificity) from a ROC curve [22]. Estimation of risk of graft loss at 24 months due to treatment or a high delta of change of eGFR was performed calculating odds ratios (OR) and their confidence intervals at 95% (CI 95%) from logistic regression models. All statistical analyses were performed using IBM SPSS statistics V20.0 software (IBM Corp, Armonk, NY, USA). Two-sided *P*-values < 0.05 were used to indicate statistical significance.

## Results

Sixty-two patients with TG (cg ≥ 1) and diagnosis of active c-aABMR according to Banff 2017 classification, were retrospectively identified and included in the study. Twenty-three received treatment with RTX, IVIG, and PE, whereas 39 did not receive additional treatment and were considered the control group. The median length of follow-up was 27 months.

In all patients but one, the indication of graft biopsy was in the context of deterioration of renal function or proteinuria (> 1 g/day). In the remaining patient, the biopsy was performed as follow-up biopsy after borderline rejection.

Table 1 summarized the demographic and clinical characteristics at the time of c-aABMR diagnosis. At the time of c-aABMR diagnosis, treated patients were significantly younger than those not treated 43.6 ± 13.2 vs. 53.6 ± 16.1 years (*P* = 0.008). However, CCI was not different between groups. In the two groups, the immunosuppressive regimens were similar (Table 1).

**Table 1:** Demographic and clinical characteristics

	Treatment (*N* = 23)	Control (*N* = 39)	*P* value
Dialysis vintage (months)	32.6 ± 24.2	41.3 ± 38.7	0.37
Donor Age	43.05 ± 15.69	50.89 ± 11.99	0.008
Donor type (Living donor)	9 (39.1%)	12 (30.7%)	0.5
First kidney transplant	13 (56.5%)	27 (69.2%)	0.31
HLA mistmatch (A, B, DR)	3.27 ± 1.1	3.8 ± 1.5	0.12
Desensitization (PE + RTX)	1 (4.34%)	1 (2.56%)	0.7
Induction (Yes)	16 (69.56%)	30 (76.92%)	0.52
Thymoglobulin / ATG	10 (43.48%)	15 (38.46%)	0.7
Basiliximab	3 (13.04%)	13 (33.3%)	0.8
Other	3 (13.04%)	2 (5.12%)	0.3
IS at time of transplantation n (%)			
Tacrolimus + MMF/MPA + PDN	12 (52.2%)	24 (58.9%)	
Cyclosporine + MMF/MPA + PDN	3 (13.04%)	5 (12.82%)	
Cyclosporine + PDN	5 (21.74%)	3 (7.69%)	
mTORi + MMF/MPA + PDN	1 (4.35%)	5 (12.82%)	
Tacrolimus + mTORi + PDN	1 (4.35%)	1 (2.56%)	
Cyclosporine + mTORi + PDN		1 (2.56%)	
Cyclosporine + Azathioprine + PDN	1 (4.35%)		
Previous treated rejections of this allograft			
Cellular rejection	6 (26.1%)	10 (25%)	0.97
Humoral rejection	7 (30.4%)	6 (15.38%)	0.26
At the time of c-aABMR diagnosis			
Sex (Female/Male)	8/15	14/25	0.92
Age (years)	43.59 ± 13.2	53.6 ± 16.1	0.013
Charlson comorbidity index (CCI)	0.83 ± 1.1	0.97 ± 1.27	0.7
Time KT to active c-aABMR (months)	92.2 ± 75	93.3 ± 55.1	0.67
eGFR (mL/min) at c-aABMR diagnosis	30.9 ± 13.5	33.4 ± 11.6	0.45
eGFR (mL/min) 6 months before cABMR	40 ± 11	42.9 ± 10.2	0.3
Proteinuria (mg/g) at c-aABMR diagnosis	2286 ± 2248	1763 ± 1427	0.31
DSA (+)	6 /9	3 / 11	0.17
Anti-HLA Antibodies (+)	13 / 16	19 / 37	0.041
IS at time of c-aABMR diagnosis n (%)			
PDN + other IS	17 (73.9%)	22 (55%)	
Tacrolimus + MMF/MPA ± PDN	9 (39.1%)	17 (42.5%)	
mTORi + MMF/MPA ± PDN	2 (8.69%)	7 (17.5%)	
Cyclosporine + MMF/MPA ± PDN	4 (17.39%)	6 (15%)	
Tacrolimus + PDN	3 (13.04%)	4 (10%)	
MMF/MPA + PDN	1 (4.34%)	3 (7.5%)	
Cyclosporine ± PDN	2 (8.69%)	2 (5%)	
Tacrolimus + mTORi ± PDN	1 (4.34%)	1 (2.5%)	
Cyclosporine + mTORi	1 (4.34%)		

Results are shown as mean ± SD or absolute frequencies (%) for quantitative and qualitative variables respectively. *GFR* glomerular filtrate rate, *KT* kidney transplant, *IS* immunossupression, *mTORi* mammalian target of rapamycin inhibitor, *MMF/MPA* mycophenolate mofetil or mycophenolic acid, *cABMR* chronic antibody-mediated rejection; PDN, prednisone, *RTX* Rituximab

Type of donor and prior kidney transplants were not statistically different between both groups (Table 1). Mean donor age was lower in the treated group 43.05 ± 15.69 vs. 50.89 ± 11.99 years (*P* = 0.035).

### Treatment

In all treated patients RTX was initiated between one and 3 weeks after c-aABMR diagnosis. 82.6% of patients treated with RTX received two doses with a mean cumulative dose of 1008 ± 342 mg.

The mean number of PE sessions was 5.8 ± 0.38, and mean total processed volume was 24.2 ± 5.4 L. The dose of IVIG was 200 mg/kg, after every second PE.

In the control group, six patients presented concomitant acute cellular rejection and were treated with corticoids.

**Fig. 1:**
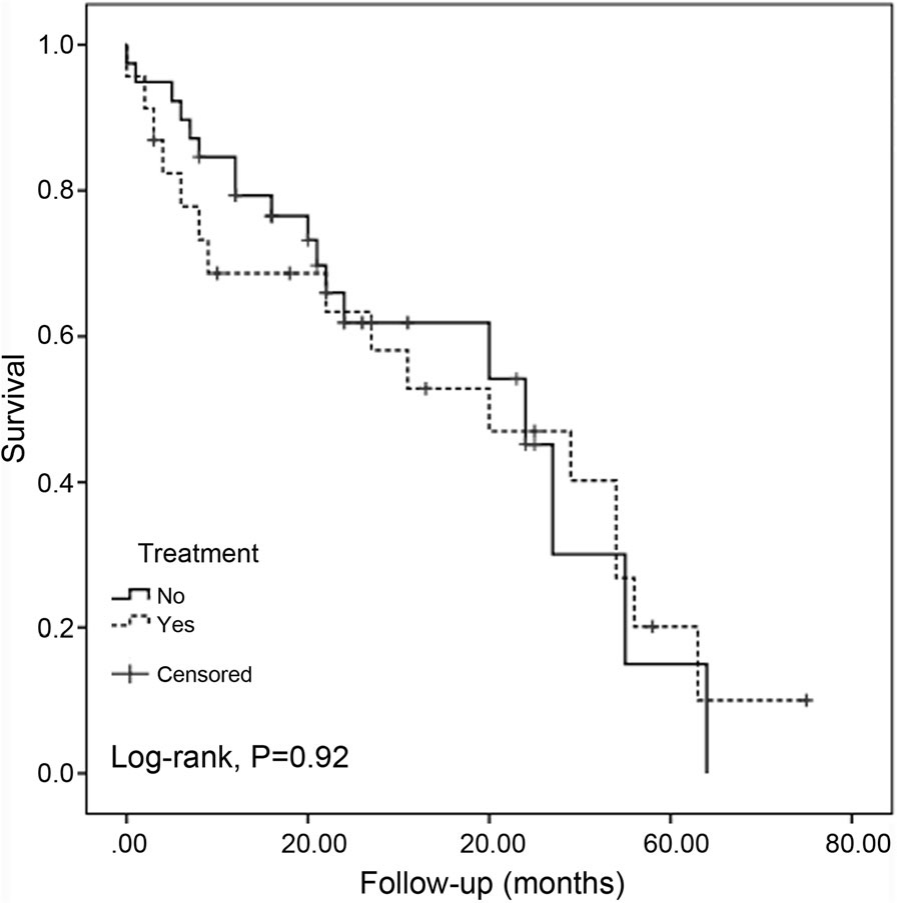
Renal allograft survival censoring death after c-aABMR diagnose. Treatment: patients under rituximab-containing treatment (yes), control patient group (no). Chronic active antibody-mediated rejection (c-aABMR)

Graft survival censoring death was not different between both groups (Fig. 1), Log Rank *P* = 0.92. The proportion of graft loss at 12 and 24 months after c-aABMR diagnosis in treated and control groups was not statistically significant, 7 patients (30%) vs. 8 patients (34.7%) and 8 (34.7%) vs.13 (33.3%) respectively.

### Patient survival

Four patients died in the treated group, two of sepsis (10 and 45 months after the initiation of treatment) and two of sudden death at home (3 and 64 months after c-aABMR treatment). None of the patients in the control group died.

### Kidney function, proteinuria, and presence of DSA

**Fig. 2:**
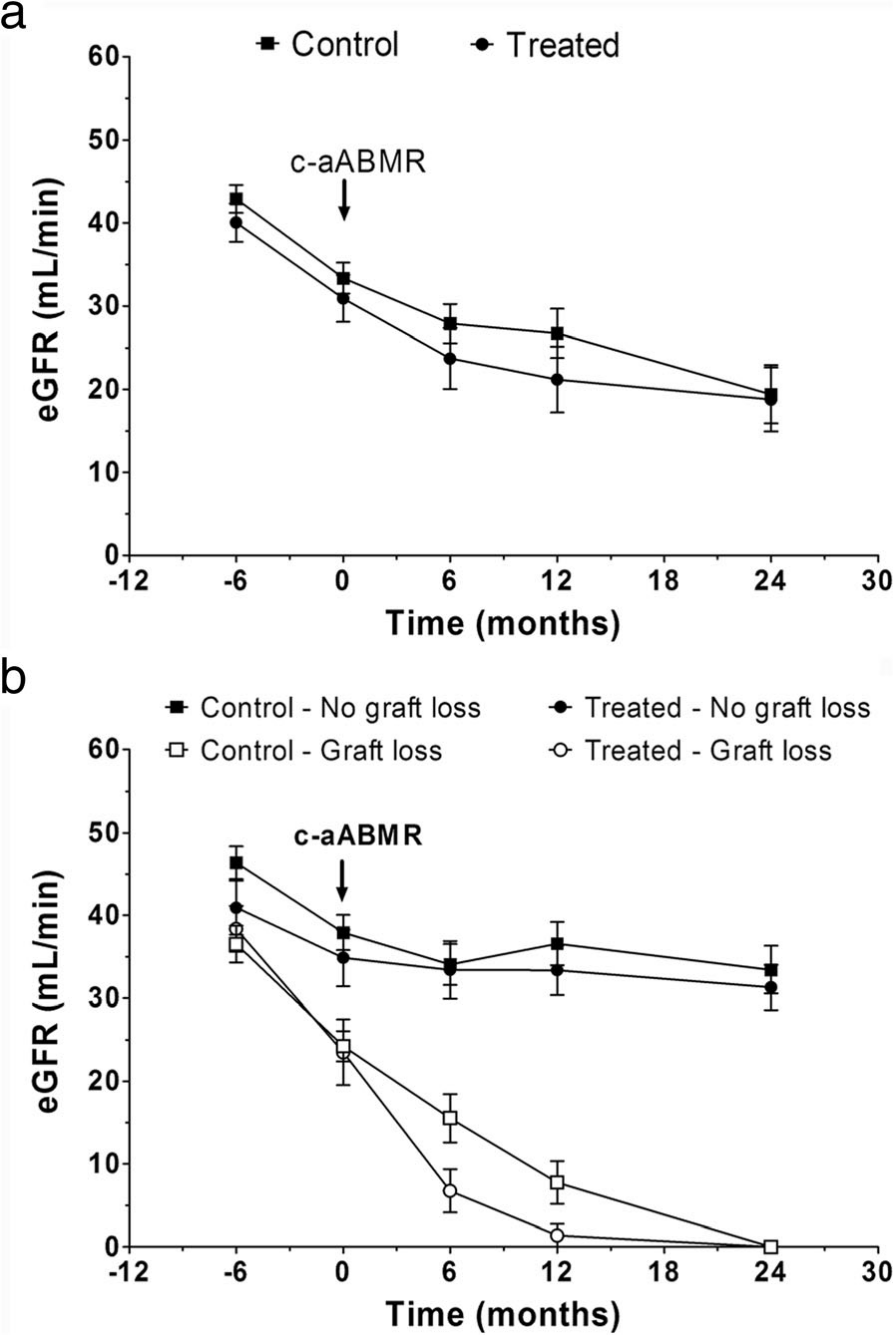
Estimated glomerular filtrate rate (eGFR) follow-up before and after c-aABMR diagnose. **a** eGFR evolution of treated and control patient groups. **b** eGFR evolution according to graft outcome in both groups. Chronic active antibody-mediated rejection (c-aABMR)

The mean eGFR at diagnosis of c-aABMR and 6 months before was not different between groups (Table 1 and Fig. 2a). Also, proteinuria at diagnosis was similar in both groups (Table 1).

The mean eGFR at 6, 12 and 24 months in treated and control patients was not different (Fig. 2a). Even if we split according to graft outcome eGFR follow-up was similar between treated and control patient. (Figs 2b and Additional file 1: Figure S1).

An elevated ΔeGFR (eGFR 6 months before diagnosis – eGFR at diagnosis of cABMR) was related to graft loss during the first 24 months after diagnosis. A proposed cut of 13 ml/min in ΔeGFR was obtained from ROC analysis with LR^+^ = 3.34. A decrease of eGRF of 13 ml/min or more was an independent indicator of graft loss in the first 24 months with OR = 5 (95% CI = 1.5–16.9; *P* = 0.01). The impact of ΔeGFR was influenced neither in magnitude or statistical significance in a multivariate approach, adjusted by treatment (Table 2). Also proteinuria higher than 2.5 g/day (LR^+^ = 3.6), adjusted for treatment, was associated with loss of graft at 24 months in both groups (OR = 3; 95% CI = 1.22–7.37; *P* = 0.016).

**Table 2:** Risk of graft loss at 24 months according to ΔeGFR and treatment

	OR & (95% CI)	*P* value	Model
Change in ΔeGFR > 13 ml/min	5 (1.5 – 16.9)	0.006	Univariate
Treatment	1.2 (0.4 - 3.5)	0.736	Univariate
Change in ΔeGFR > 13 ml/min	5 (1.5 – 16.9)	0.006	Multivariate
Treatment	1.1 (0.3 - 3.4)	0.897	

*ΔeGFR* eGFR six months before diagnosis - eGFR at diagnosis of c-aABMR

**Fig. 3:**
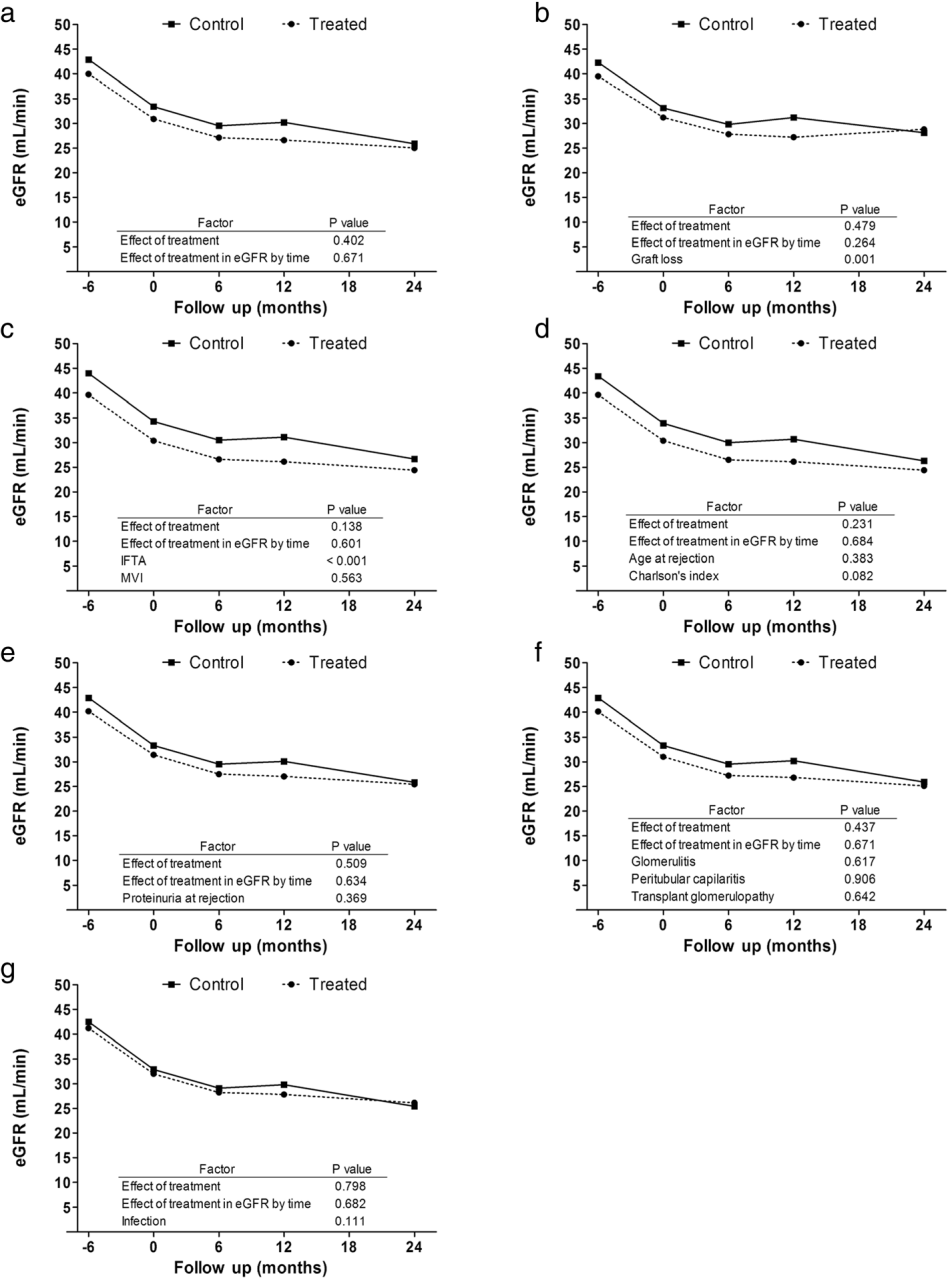
Crude and adjusted estimation of eGFR according to GEE longitudinal models. Crude model (**a**); adjusted by graft loss (**b**); adjusted by IFTA (interstitial fibrosis and tubular atrophy) and MVI (microvascular inflammation) (**c**); adjusted by age and Charlson comorbidity index (**d**); adjusted by proteinuria (**e**); adjusted by glomerulitis, capillaritis and transplant glomerulopathy (**f**); adjusted by infection disease complications in the follow-up (**g**). Estimated glomerular filtrate rate (eGFR)

We have evaluated the impact of treatment on the deterioration of renal function in a longitudinal model analysis (Fig. 3), showing that the impairment of eGFR is independent of the treatment in a crude estimation model. Also, we evaluated the impact of treatment adjusted by possible confounding factors such as age, Charlson’s index, graft loss, IFTA, microvascular inflammation (MVI), transplant glomerulopathy (TG), glomerulitis and peritubular capillaritis scores, proteinuria or presence of infections. However, the decrease in eGFR remains independent of the treatment (Fig. 3).

Proteinuria was not different in both groups at 6 and 12 months, mean 1.8 ± 1.2 vs. 1.7 ± 1.5 and 1.7 ± 1 vs. 1.8 ± 1.8 g/g (*P* = 0.9) creatinine respectively.

Anti-HLA antibodies were more prevalent in the treated patients, but no difference was observed in de novo DSA prevalence (*P* = 0.03 and 0.17 respectively). Regarding the DSA class, 22% were class II, 33,3% class I and, 44,4% class I and II.

The positivity of DSA and anti-HLA antibodies were not associated with worse graft survival at 24 months in our series (*P* = 0.06 and 0.65 respectively).

### Histological features

The time between renal transplantation and graft biopsy was similar in treated and control patients, 92.2 ± 75 and 93.3 ± 55 months respectively (*P* = 0.94).

At diagnosis, histologically acute inflammatory and chronic lesions related to c-aABMR and TG were similar in both groups (Table 3). Transplant glomerulopathy and IFTA were similar in treated and control patients, 1.74 ± 0.83 vs. 1.83 ± 0.77 (*P* = 0.54) and 1.61 ± 0.78 vs. 1.83 ± 0.84 (*P* = 0.27) respectively. The presence of IFTA ≥ two was associated with graft loss at 24 months, (OR = 4.7; 1.19–18.5; *P* = 0.02).

**Table 3:** Banff histopathological features at diagnosis of cABMR

	Treatment (*N*=23)	Control (*N*=39)	*P* value
MVI (g+tc)	2.78 ± 1.35	2.87 ± 1.36	0.67
i	0.6 ± 0.78	0.56 ± 0.65	0.95
t	0.17 ± 0.49	0.25 ± 0.69	0.97
g	1.52 ± 0.94	1.4± 0.94	0.63
ptc	1.26 ± 0.86	1.4 ± 0.79	0.4
ah	1.43 ± 1.04	1.38 ± 1.1	0.91
cg	1.74 ± 0.83	1.83 ± 0.77	0.54
ci	1.52 ± 0.79	1.83 ± 0.88	0.17
ct	1.56 ± 0.73	1.67 ± 0.89	0.62
IFTA	1.61 ± 0.78	1.83 ± 0.84	0.27
cv	1.17 ± 0.83	1.39 ± 0.87	0.46
C4d deposition	19 (82.6%)	17 (43.6%)	0.01
acute cellular rejection	0	6	0.05

Results are shown as mean ± SD or absolute frequencies (%) for quantitative and qualitative variables respectively. *i* interstitial inflammation, *t* tubulitis, *g* glomerulitis, *ptc* peritubular capillaritis, *ah* arterial hyalinosis, *cg* transplant glomerulopathy, *ci* interstitial fibrosis, *ct* tubular atrophy, *IFTA* interstitial fibrosis + tubular atrophy, *cv* vascular fibrous intimal thickening

C4d deposition was more frequent in treated patients 19 (82.6%) vs. 17 (43.6%) (*P* = 0.001). The positivity of C4d, the severity of MVI (g + ptc) and TG degree were not associated with graft loss or worse survival.

Ten of the 23 treated patients had post-treatment follow-up biopsies within the first year after treatment with persisting active c-aABMR in all ten biopsies.

### Adverse events

Infections that required hospitalization at least 48 h were more common in treated than non-treated patients during the first year after c-aABMR diagnosis, 15 vs. 8 (OR = 4.22; 95% CI = 1.37–13.1; *P* = 0.012), this reflects a ratio of infection/patient per year of 0.65 and 0.25 in treated and control patients respectively.

Infections were respiratory (8), urinary tract (6), cutaneous and mucosal (3), abdominal (4), disseminated zoster (1), and sepsis (1). The microbiological isolation was negative in 12 cases, 3 *Pseudomonas aeruginosa*, 2 *Cytomegalovirus*, 1 *Klebsiella pneumoniae*, 1 *Escherichia coli*, 1 *Enterococcus faecalis*, 1 *Campylobacter jejuni* and 1 *Herpes Zoster* virus.

A CCI of 3 was associated with more infectious complications in the control group (OR = 8.7; 95% CI = 1.15–65.9; *P* = 0.036), but not in the treated patients (*P* = 0.16). Related to adverse reactions in PE sessions or RTX infusion, only one patient developed tetany related to hypocalcemia.

## Discussion

In this study, treatment with rituximab + IVIG and PE was not associated with improved graft survival when compared with the control group. On the other hand, the incidence of infections requiring hospitalization within 1 year after treatment was more than doubled in the treated group.

Chronic antibody-mediated damage is the main limitation for long-term graft survival, but currently, only scarce data are available about the treatment of active c-aABMR with TG. In small retrospective series of cases, the partial effectiveness of RTX and IVIG has been reported [7–10]. Rostaing et al. reported 14 patients with TG treated with RTX and steroids showing stabilization or improvement of renal function in seven patients. Four patients (28.5%) presented severe infections in the first year after treatment [23].

A prospective study in 20 pediatric patients with c-aABMR treated with one dose of RTX and a high dose of IVIG reported good response in all the patients without TG, but only in 45% of the patients with TG [10]. The response was defined as a reduction in the decline of GFR of 30%.

In another retrospective series of 31 patients with c-aABMR and TG treated with RTX (*n* = 14) vs. no treatment (*n* = 17), only eight patients responded to treatment [24]. The response was defined as a decline or stabilization of serum creatinine for at least 1 year.

These studies highlight the importance of TG as a marker of chronic damage and a poor prognosis. On the other hand, efficacy was based on graft function stabilization, which is difficult to distinguish from the natural history of the disease in the absence of an untreated control group. Indeed, in our control group, some patients stabilized their renal function. As in other reported series, the evolution of these patients is heterogeneous in both groups, which highlights the importance of having a control group in future studies.

Other recent studies reported that RTX treatment did not improve graft survival compared to an untreated group. Moreover, a higher incidence of adverse effects was detected in the treatment groups [11, 12]. However, the untreated groups were small in both studies, and they did not evaluate the combination of RTX, PE, and IVIG.

We performed the analysis using two comparable cohorts. Also, c-aABMR treatment was homogeneous.

The decision to treat was based on individual clinical judgment and seems to be influenced by the perception of a better performance status of the patient, younger age, younger donor and less risk of infections. However, even with this potential positive selection bias, severe infections were more frequent in treated patients than in the older control group. In fact, the CCI was similar in both groups but was only associated with more infections in the control patients.

On the contrary with results presented in other studies [10], the severity of transplant glomerulopathy, (cg) score, was not associated with worse survival or loss of the graft at 24 months. Probably TG indicates a late non-reversible manifestation of antibody-mediated processes. Ten patients had a control biopsy after treatment, none presented improvement of TG.

In contrast with our data Kahwaji et al. suggest that patients with a high ptc and MVI scores may benefit from treatment with IVIG and RTX. But this was a trend that was not statistically significant [12]. Similar to our findings the authors did not find C4d positivity to be associated with worse graft outcomes, which is in contrast to the previous reports [5, 25–29].

A low eGFR at diagnosis is associated with graft loss at 24 months [11, 12]. We postulate that ΔeGFR, between 6 months before rejection and the time of diagnosis of rejection, ≥13 ml/min is more helpful to identify the patients with worse graft survival at 24 months.

In an observational study, 114 consecutive kidney transplant patients with c-ABMR were treated with steroids and IVIG. Three-fourths of patients lost their kidney grafts with a median survival of 1.9 years. The addition of rituximab or thymoglobulin in 40% of patients did not improve graft survival [30].

Recently a Spanish multicenter randomized trial has been performed in order to analyze the efficacy of rituximab and IVIG vs. placebo in 24 patients with TG and DSA positivity (12 placeboes vs. 12 treatments) [13]. The primary outcome was the difference in the decline of eGFR at 12 months. In concordance with our study, there was no difference in eGFR decline. Unfortunately, the study was stopped, after recruiting only 50% of the minimal sample calculated, due to low inclusion rate, and was underpowered, thus, highlighting the difficulty for prospective studies in this area.

Another strategy includes the use of bortezomib, a proteasome inhibitor. Recently a randomized trial has been presented comparing bortezomib treatment vs. placebo in late ABMR, which included 28 patients with cABMR. Bortezomib treatment failed to induce a reversal in decline of eGFR, DSA changes or morphologic and molecular features of disease activity in follow-up biopsies. In this trial, treatment was associated with substantial toxicity [31].

Given the poor results and the higher incidence of infectious complications, the unmet need is to improve diagnosis and enhance treatment options. Use of electron microscopy to detect early forms of TG (cg = 1a) or increased expression of gene transcripts indicative of endothelial injury might be helpful to improve graft survival.

New therapeutic options with more potent and less toxic immunosuppressive drugs or alternative immunological interventions are required. In this regard, two small prospective studies evaluated the complement system blockade at different levels in the treatment and prevention of c-aABMR and TG, without changes in long-term outcomes [32, 33].

In another recent report, Tocilizumab (Anti-IL6 receptor monoclonal antibody) showed promising results with stabilization of renal function in a small series of patients with c-aABMR and TG [34].

A significant cause of DSA and c-aABMR development is non-adherence to immunosuppression therapy. In this context, it seems to be more efficient and less dangerous to focus on promoting immunosuppressant therapy adherence rather than treating c-aABMR with TG aggressively.

This study presents a large group of patients with uniform pathology and treatment. However, the retrospective nature of the study is a limitation. DSA, and anti-HLA description is incomplete, hypogammaglobulinemia was not recorded and the dose of IVIG was low. In spite of the impossibility to assess the presence of DSA in all patients, the failure to demonstrate DSA does not rule out its existence [35]. The Banff´17 classification recognizes the fact that current DSA testing methods do not detect all antibodies that are potentially injurious to the allograft, and recommends the use of alternative markers that are not available in our center [3]. Recently, Sablik et al. analyzed whether cases suspicious for c-aABMR (DSA negative, *n* = 24) differ from cases of c-aABMR (DSA positive, *n* = 17) with respect to renal histology, allograft function and long-term graft survival [36]. There were no statistically significant differences on the decline of allograft function and renal allograft survival in cases with or without DSAs.

On the other hand, a strength of this study is that it shows a realistic incidence of serious infectious complications after treatment that should be taken into account in the therapeutic decision.

## Conclusions

In summary, the rapid decline in GFR between 6 months before rejection diagnosis and the time of diagnosis is associated with poor prognosis. Treatment with RTX, PE, and IVIG in patients with active c-aABMR with TG was not associated with better graft survival, but a significant increase in serious infectious complications was observed.

## Additional file

Additional file 1: **Figure S1.** Individual eGFR follow-up. (**a-b**) eGFR evolution of control and treated patients without graft loss. (**c-d**) eGFR evolution of control and treated patients with graft loss. eGFR, estimated glomerular filtrate rate; c-aABMR, chronic active antibody-mediated rejection (DOCX 444 kb)

## Abbreviations

c-aABMR: Chronic active antibody-mediated rejection
CCI: Charlson comorbidity index
DSA: Donor specific antibodies
eGFR: Estimated glomerular filtrated rate
IFTA: Interstitial fibrosis and tubular atrophy
IVIG: Intravenous immunoglobulin
MMF/MPA: Mycophenolate mofetil or mycophenolic acid
MVI: Microvascular inflammation
PDN: Prednisone
PE: Plasma exchange
RTX: Rituximab
TG: Transplant glomerulopathy
