# Supplementary material for: Rituximab, plasma exchange and immunoglobulins: an ineffective treatment for chronic active antibody-mediated rejection
Source: BMC Nephrol. 2018 Oct 11;19:261. doi: 10.1186/s12882-018-1057-4 (PMC6182805; doi:10.1186/s12882-018-1057-4)
Supplement: Supplementary file 1 — Figure S1. Individual eGFR follow-up. (a-b) eGFR evolution of control and treated patients without graft loss. (c-d) eGFR evolution of control and treated patients with graft loss. eGFR, estimated glomerular filtrate rate; c-aABMR, chronic active antibody-mediated rejection (DOCX 444 kb) [file 12882_2018_1057_MOESM1_ESM.docx]

**Supplementary Material**

**
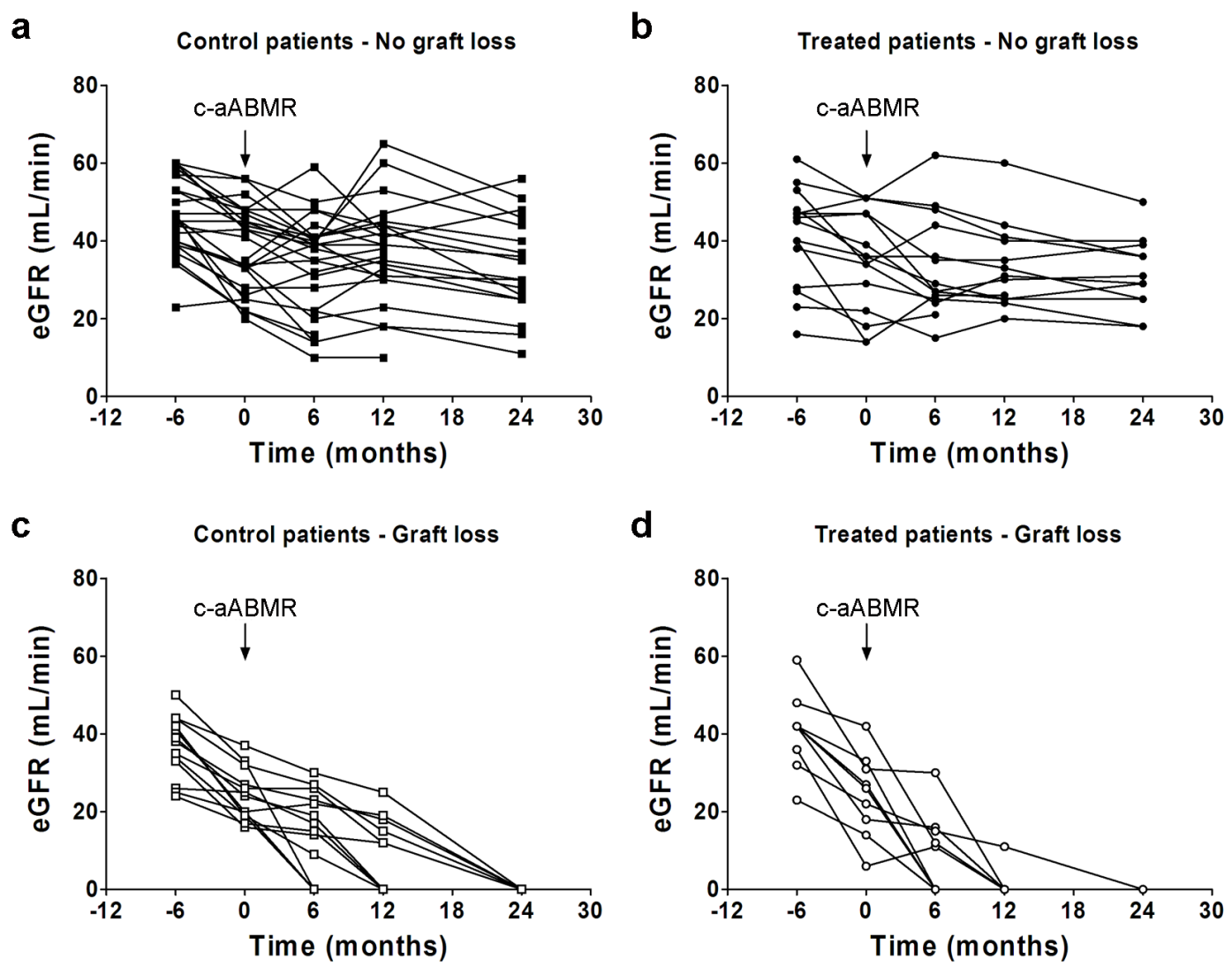
**

**S1 Fig. Individual eGFR follow-up.** (**a-b**) eGFR evolution of control and treated patients without graft loss. (**c-d**) eGFR evolution of control and treated patients with graft loss. eGFR, estimated glomerular filtrate rate; c-aABMR, chronic active antibody-mediated rejection.
